# Vacquinol-1 inducible cell death in glioblastoma multiforme is counter regulated by TRPM7 activity induced by exogenous ATP

**DOI:** 10.18632/oncotarget.16703

**Published:** 2017-03-30

**Authors:** Philip Sander, Haouraa Mostafa, Ayman Soboh, Julian M. Schneider, Andrej Pala, Ann-Kathrin Baron, Barbara Moepps, C. Rainer Wirtz, Michael Georgieff, Marion Schneider

**Affiliations:** ^1^ Division of Experimental Anesthesiology, University Hospital Ulm, 89081 Ulm, Germany; ^2^ Department of Neurosurgery, Bezirkskrankenhaus Guenzburg, 89312 Guenzburg, Germany; ^3^ Department of Operative Dentistry and Periodontology, University Hospital Ulm, 89081 Ulm, Germany; ^4^ Institute of Pharmacology and Toxicology, University Hospital Ulm, 89081 Ulm, Germany; ^5^ Department of Anesthesiology, University Hospital Ulm, 89081 Ulm, Germany

**Keywords:** glioblastoma multiforme, Vacquinol-1, ATP, TRPM7, methuosis

## Abstract

Glioblastomas (GBM) are the most malignant brain tumors in humans and have a very poor prognosis. New therapeutic options are urgently needed. A novel drug, Vacquinol-1 (Vac), a quinolone derivative, displays promising properties by inducing rapid cell death in GBM but not in non-transformed tissues. Features of this type of cell death are compatible with a process termed methuosis. Here we tested Vac on a highly malignant glioma cell line observed by long-term video microscopy. Human dental-pulp stem cells (DPSCs) served as controls. A major finding was that an exogenous ATP concentration of as little as 1 μM counter regulated the Vac-induced cell death. Studies using carvacrol, an inhibitor of transient receptor potential cation channel, subfamily M, member 7 (TRPM7), demonstrated that the ATP-inducible inhibitory effect is likely to be via TRPM7. Exogenous ATP is of relevance in GBM with large necrotic areas. Our results support the use of GBM cultures with different grades of malignancy to address their sensitivity to methuosis. The video-microscopy approach presented here allows decoding of signaling pathways as well as mechanisms of chemotherapeutic resistance by long-term observation. Before implementing Vac as a novel therapeutic drug in GBM, cells from each individual patient need to be assessed for their ATP sensitivity. In summary, the current investigation supports the concept of methuosis, described as non-apoptotic cell death and a promising approach for GBM treatment. Tissue-resident ATP/necrosis may interfere with this cell-death pathway but can be overcome by a natural compound, carvacrol that even penetrates the blood-brain barrier.

## INTRODUCTION

Approximately 30% of all primary brain tumors are diagnosed as Glioblastoma multiforme (GBM), a highly fatal malignancy with high mutagenicity and extreme migration and invasion potentials, explaining the poor survival rate [1]. In addition to standard radiochemotherapy inducing apoptosis-related cell death, new effective therapeutic modalities for GBM are urgently required. Established apoptosis-directed therapy has not resulted in an improved prognosis [2]. Importantly, GBM disseminate within the brain and lack hematogenous spreading [3–5]. Nevertheless, major changes of the patients’ immune system are consistently found [6], implying an as yet not fully understood crosstalk between the tumor environment and hematological/immunological elements. Alterations of the immune system are relevant to survival [6, 7].

Only recently, screening of a small molecule library (NCI-DTP Diversity Set II) revealed a quinolone substance termed Vacquinol-1 (Vac), which induces a novel form of a cell death that appears to be highly restricted to GBM [8]. Characteristics of Vac-induced glioma cell death include cell blebbing followed by rupture of the plasma membrane. *In situ*, GBMs contain large areas of necrotic tissue [9, 10], which are likely to be ATP-rich [11]. Extracellular ATP is an important signaling molecule that acts via purinergic receptors and ion channels [12, 13]. Moreover, extracellular ATP possesses a growth-promoting effect on glioma cells [14], and may act through metabotropic P2Y and ionotropic P2X receptors [12]. Therefore, we considered whether ATP might influence Vac-inducible cell death. In addition to purinergic receptors, ATP can lead to activation of the ubiquitously expressed transient receptor potential cation channel, subfamily M, member 7 (TRPM7) cation channel [15]. TRPM7 plays a role in several cancer types, including breast cancer [16], gastric cancer [17] and pancreatic cancer [18]. The high physiological relevance of TRPM7 for GBM was recently demonstrated by the remarkable growth-inhibitory effect when TRPM7 was blocked [19]. Chen and colleagues demonstrated that the tumoricidal activity of the natural terpenoid carvacrol was through TRPM7 inhibition [19]. In this study, we addressed Vac-inducible cell death in an extremely aggressive glioblastoma cell line (#12537-GB) and its sensitivity to ATP using long-term observation by live cell imaging (IncuCyteZOOM®, Essenbio.com, USA). Results provide evidence for i) Vac-induced cell death in GBM-derived cell lines, but not in dental-pulp stem cells (DPSCs), and ii) the potential of ATP-induced counter regulation. The ATP-related effects appear to be mediated by TRPM7 and are sensitive to carvacrol. One important rationale to address ATP induced effects arises from its properties to function as a danger signal in solid tumors [20].

## RESULTS

### Vac induces rapid cell death in glioma cell lines and is sensitive to exogenous ATP

**Figure 1:**
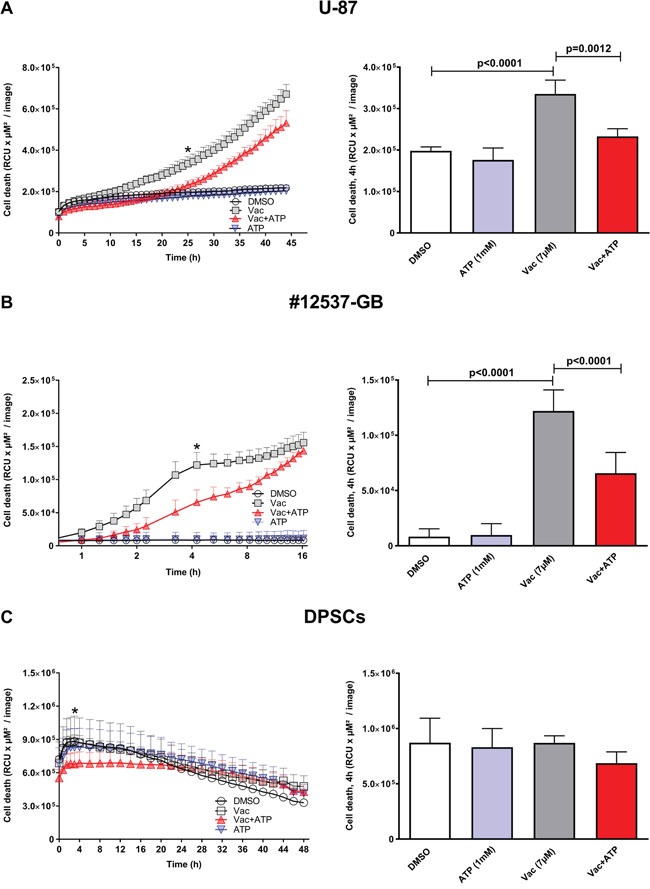
Vac efficiently kills glioma cells in an ATP-sensitive manner, but does not affect DPSCs Kinetics of cell death were determined from the PI-positive (dead) cells displayed as relative color units (RCU) per μM^2^/image (y-axis, left column). U-87 cells were followed for 45 h (x-axis) (IncuCyteZOOM®). DMSO diluted in medium at 10^−4^ served as control; 7 μM Vac; Vac+ATP 1 mM; and 1 mM ATP alone (**A, left**). Quantitative analysis of cell death determined at 25 h (*) was significantly different for Vac vs. control (multiple comparison two-way ANOVA, p<0.0001) and Vac vs. Vac+ATP (multiple comparison two-way ANOVA, p=0.0012) (**A, right**). Cell death of #12537-GB cells was followed for 16 h (x-axis, log2 scale). DMSO (10^−4^) control; 7 μM Vac; Vac+ATP 1 mM; and 1 mM ATP alone (**B, left**). Quantitative analysis at 4 h (*) was significantly different for Vac vs. control and Vac vs. Vac+ATP (both p<0.0001 by multiple comparison two-way ANOVA) (**B, right**). Kinetics of cell death determined by PI-positive (dead) DPSCs (RCU×μM^2^/image) were followed for 48 h (IncuCyteZOOM®). DMSO (10^−4^) control; 7 μM Vac; Vac+ATP 1 mM; and 1 mM ATP alone (**C, left**). Quantitative analysis at 4 h (*) for DPSCs (**C, right**). All values are given as means ± SD (triplicate values). The analyses were performed with 5,000 cells seeded per well on day 1 in 96-well flat-bottomed microtiter plates. All imaging was performed using IncuCyteZOOM® at 10× objective. Results are representative of 3 independent experiments.

In the present study, we investigated the kinetics of Vac on the viability of the established glioma cell line U-87 and the new, highly aggressive glioma cell line designated #12537-GB, and thus confirmed and extended endpoint observations reported by Kitambi and colleagues [8]. To determine cell viability of pre-established cell layers, we applied the live cell imaging system IncuCyteZOOM® equipped with a 10× objective and used propidium iodide (PI) staining to determine cell death. Vac concentrations of 0.07 μM and 0.7 μM were applied to #12537-GB, but there was no significant effect on cell viability (IncuCyteZOOM®, kinetics of cell death for 43 h). With high Vac concentrations (14 μM, 28 μM), glioma cells died in <2 h (Supplementary Figure 1). For further experiments, we chose a Vac concentration of 7 μM for all kinetic analyses. Figure 1 shows the results of continuous imaging of both phase contrast and red fluorescence over a period of 16 h (#12537-GB), 45 h (U-87), or 48 h (DPSCs), with frames taken at 15–120-min intervals. Red fluorescence corresponding to dead cells ranged between 2–5% of the total population in U-87 and #12537-GB control cell layers (Figure 1A, 1B). When trying to interfere with Vac-induced cell death, we identified that exogenous ATP exhibited the most pronounced counter regulatory effect (Figure 1A, 1B). Within 1.25 h of observation, Vac treatment of the #12537-GB cell line already differed significantly in viability from untreated controls (two-way analysis of variance (ANOVA), p<0.0001), Figure 1A). In contrast, viability impaired by Vac treatment of U-87 only differed at 8 h from untreated controls (Figure 1B). The time point of greatest differences between Vac and Vac+ATP treatments is illustrated in the right columns of Figure 1. After 4 h of incubation, the differences in PI fluorescence corresponding to cell death were significant in #12537-GB (multiple comparison two-way ANOVA, p<0.0001: control/Vac; p<0.0001: Vac/Vac+ATP). However, in U-87, the time point of greatest difference was observed later, at 25 h of incubation (multiple comparison two-way ANOVA, p<0.0001: control/Vac; p=0.0012: Vac/Vac+ATP). The results were compared with DPSCs, a stem cell line from third molars (Figure 1C). Vac-treated DPSCs did not display any significant PI uptake during the observational period (Figure 1C). The right column of Figure 1C illustrates PI uptake after 4 h of incubation in DPSCs, which was not significant after treatment. Taken together, Vac-induced cell death is sensitive to the counter regulatory effect by ATP, following cell-specific kinetics: Vac induced almost 100% cell death in #12537-GB within 16 h of incubation whereas U-87 cells responded in a delayed fashion, with cell death still on-going after 45 h of incubation.

### Vac-induced cell death coinciding caspase 3/7 activation is counter regulated by exogenous ATP in glioma cell lines

**Figure 2:**
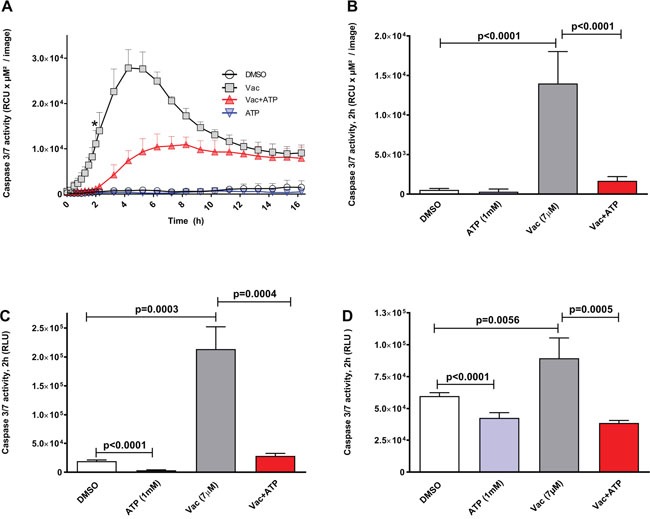
Vac leads to caspase 3/7 activation in glioma cells, counter regulated by ATP Caspase 3/7 activity given as relative color units (RCU) per μM^2^/image. Semi-confluent glioma cells (seeded in 96-well flat-bottomed microtiter plates) were treated with DMSO diluted in medium at 10^−4^ served as control; 7 μM Vac; Vac+ATP 1 mM; and 1 mM ATP alone. Caspase 3/7 activity was monitored over 16 h (x-axis) (IncuCyteZOOM®) by adding IncuCyte® Caspase-3/7 reagent (y-axis, RCU×μM^2^/image) **(A)**. Quantitative analysis at 2 h for glioma cells (* in A). Vac vs. control and Vac vs. Vac+ATP (both p<0.0001 by multiple comparison two-way ANOVA). **(B)**. All values are given as means ± SD (triplicate values). All imaging was performed using IncuCyteZOOM® at 10× objective. Caspase 3/7 activity (Promega.com) in #12537-GB at 2 h of incubation (6× replicates); RLU (y-axis) **(C)**. Vac leads to increased caspase 3/7 activity compared to controls (t-test, p=0.0003); ATP counter regulates the Vac-induced caspase 3/7 activation (t-test, p=0.0004); exogenous ATP influences spontaneous caspase 3/7 activity compared to the DMSO control (t-test, p<0.0001). Caspase 3/7 activity in U-87 determined by a luminescence-based assay (n=6) after 2 h of incubation (Caspase Glo 3/7, Promega.com) given as RLU (y-axis) with background subtracted. Vac leads to increased caspase 3/7 activity compared to the DMSO control (t-test, p=0.0056); ATP counter regulates the Vac-induced caspase 3/7 activation (t-test, p=0.0005); exogenous ATP affects spontaneous caspase 3/7 activity compared to the DMSO control (t-test, p<0.0001) **(D)**. All values are given as means ± SD (6× replicates). All analyses were performed with 5,000 cells seeded per well on day 1 in 96-well flat-bottomed microtiter plates. Results are representative of 3 independent experiments.

We then investigated whether Vac-induced cell death was related to apoptosis. Therefore, caspase 3/7 was quantified using enzyme-activity assays (Essenbio.com), generating a green fluorescent signal detectable by IncuCyteZOOM®-assisted live cell imaging over a period of 16 h (Figure 2A). Vac rapidly induced an increase in caspase 3/7 activity, reaching a maximum after 6 h (Figure 2A). Vac-induced caspase 3/7 activation is sensitive to exogenous ATP. When the two time curves were compared over the period 0–16 h, differences determined by two-way ANOVA were highly significant (p<0.0001); Figure 2B shows the significances of the differences in cell death determined after 2 h of observation using multiple comparison two-way ANOVA (p<0.0001: control/Vac; p<0.0001: Vac/Vac+ATP). In addition, caspase 3/7 activity measurements using luminescence-based endpoint caspase 3/7 assay confirmed a significant increase in caspase 3/7 activity after 2 h of incubation in #12537-GB cells (Figure 2C, t-test, p=0.0003: control/Vac) and in U-87 cells (Figure 2D, t-test, p=0.0056: control/Vac). The observed upregulation of caspase 3/7 was sensitive to 1 mM ATP in #12537-GB (Figure 2C, t-test, p=0.0004) and in U-87 (Figure 2D, t-test, p=0.0005). ATP at 1 mM also attenuated spontaneous caspase 3/7 activity in both cell lines (t-test, p<0.0001: Figure 2C, 2D).

### Vac induces cell death through caspase 3/7 activation but cell death remains morphologically distinct from apoptosis induced by staurosporine (STS)

**Figure 3:**
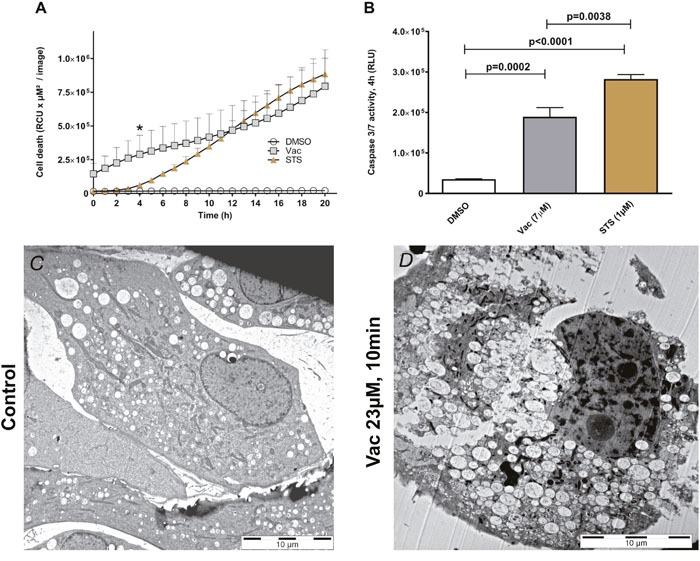
Vac- vs STS-mediated cell death Semi-confluent glioma cells (seeded in 96-well flat-bottomed microtiter plates) were treated with 2×10^−3^ DMSO diluted in medium served as control, 7 μM Vac or 1 μM STS. Cell death was monitored over 20 h (x-axis) by PI staining (y-axis, RCU×μM^2^/image). All values are given as means ± SD (8× replicates) **(A)**. All imaging was performed using IncuCyteZOOM® at 10× objective. Caspase 3/7 activity was determined by luminescence assay (RLU, y-axis by Caspase Glo 3/7 assay, Promega.com) after 4 h (*, A); control vs. Vac (t-test, p=0.0002); control vs. STS (t-test, p<0.0001); Vac vs. STS (t-test, p=0.0038). All values are means of RLU with background subtracted ± SD (8× replicates) **(B)**. Representative transmission electron micrograph image of #12537-GB without **(C)** and with 23 μM Vac **(D)** applied for 10 min at 37°C. Specimens were processed by cryo-fixation.

To compare Vac-induced cell death with typical apoptosis, cells were treated with either 7 μM Vac or 1 μM STS. STS is a potent inducer of caspase 3/7 and apoptosis [21]. Cell death was monitored over time (20 h) by PI staining and IncuCyteZOOM®-assisted live cell imaging (Figure 3A). Although both STS- and Vac-induced cell death, according to PI uptake this was delayed with STS compared to Vac (Figure 3A). Luminescence-based caspase 3/7 assays showed increased caspase 3/7 activity by STS after 4 h (t-test, p=0.0038: STS vs.Vac). To summarize, despite caspase 3/7 activation, Vac-inducible cell death at 7 μM did not lead to an apoptotic phenotype, which includes nuclear fragmentation; instead, glioblastoma cells died by vacuolization and eventual rupture of the plasma membrane (Figures 3C, 3D; Supplementary Figure 2), facilitating rapid PI uptake (Figures 1A, Supplementary Figure 2). In #12537-GB, these effects occurred earlier than in STS-induced apoptosis. Therefore, Vac-induced cell death appears to circumvent apoptosis but does involve caspase3/7 activation. Co-incubation with the pan-caspase inhibitor carbobenzoxy-valyl-alanyl-aspartyl-[O-methyl]-fluoromethylketone (zVAD-FMK) (40 μM) (Promega.com, USA) resulted in a delay of Vac-induced cell death (two-way ANOVA, p<0.0001; Supplementary Figure 3). Even in the presence of the caspase inhibitor, 100% cell death occurred later, suggesting a cooperative effect of caspase activities in Vac-induced cell death.

### Vac-induced cell death is sensitive to micromolar ATP levels

Purinergic receptors differ in ATP binding affinities (reviewed in [12]), and receptor involvement may be deduced by determination of the ATP concentration with the greatest biological effect. Of note, using flow cytometry, P2×4 and P2×7 expression was verified in #12537-GB (Supplementary Figure 4).

**Figure 4:**
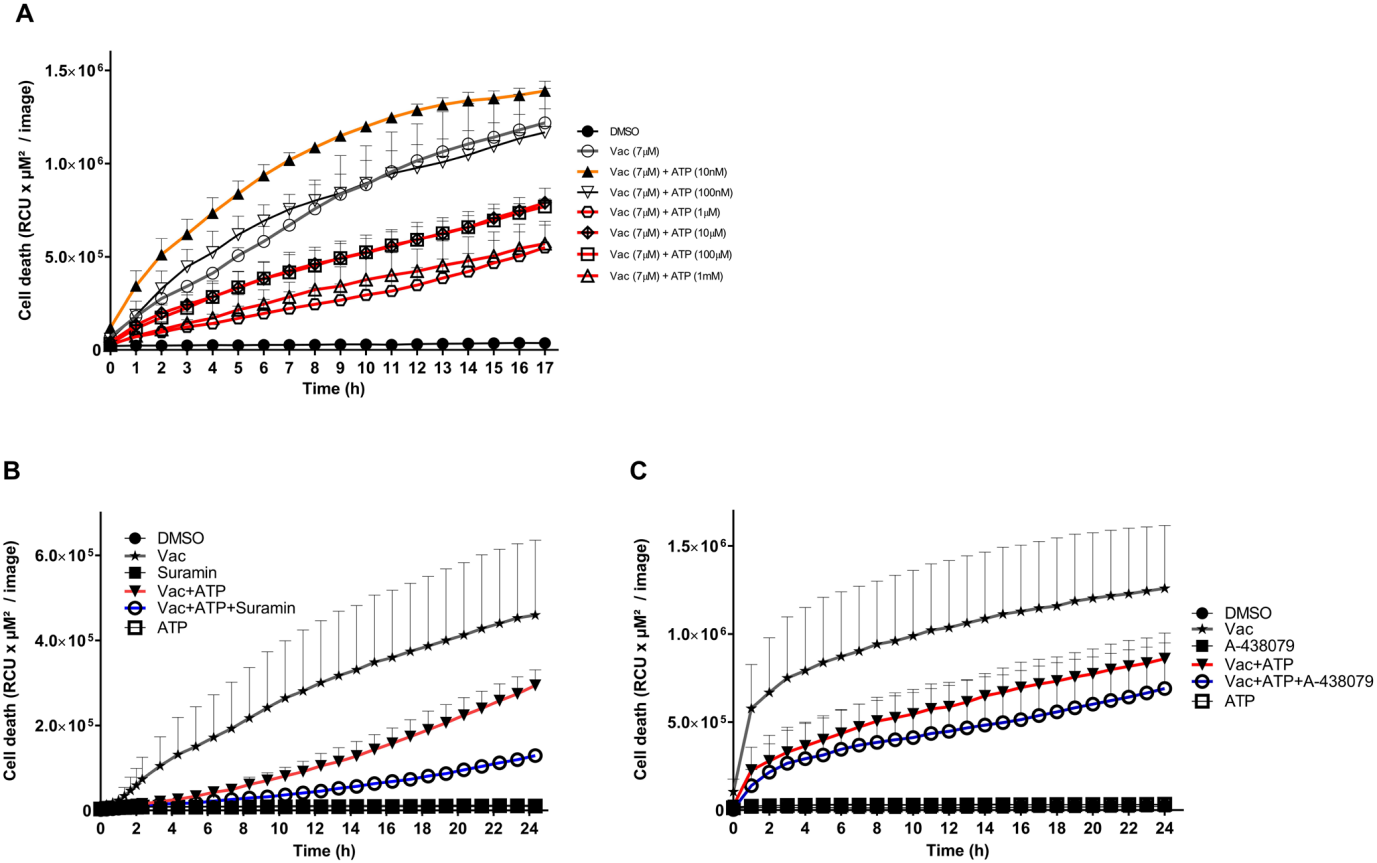
ATP concentrations interfering with Vac-induced cell death, inhibition by purinergic receptor inhibitors Semi-confluent #12537-GB cells (seeded in 96-well flat-bottomed microtiter plates) were treated with 7 μM Vac with or without 10 nM, 100 nM, 1 μM, 10 μM, 100 μM and 1 mM ATP, or DMSO diluted in medium at 10^−4^ served as control. PI-positive (dead) cells are given as RCU (y-axis, RCU×μM^2^/image). Glioma cells were followed for 17 h (x-axis). All values are means of PI fluorescence ± SD (triplicate values) **(A)**. Vac-induced cell death (7 μM) (y-axis, RCU×μM^2^/image) recorded for 24 h (x-axis) was attenuated by ATP (1 mM) but was further increased by ATP in the presence of the universal purinergic inhibitor Suramin (30 μM). All values are means of PI fluorescence ± SD (triplicate values) **(B)**. Vac-induced cell death (7 μM) (y-axis: RCU×μM^2^/image) recorded for 24 h (x-axis) was attenuated by ATP (1 mM) but was further increased by ATP in the presence of the selective P2×7 inhibitor A-438079 (100 μM). All values are means of PI fluorescence ± SD (6× replicates) **(C)**. All imaging was performed using IncuCyteZOOM® at 10× objective. Results are representative of 2 independent experiments.

Therefore, ATP was titrated between 10 nM and 1 mM into cultures with and without a constant Vac concentration of 7 μM (Figure 4A). Co-titration of 1 μM, 10 μM, 100 μM and 1 mM ATP led to significantly reduced PI uptake compared to Vac alone (two-way ANOVA, p<0.0001). By contrast, 100 nM ATP had no effect on Vac-induced cell death, whereas as little as 10 nM ATP resulted in a significant increase of Vac-induced cell death (two-way ANOVA, p<0.0001; Figure 4A). Multiple ATP-binding receptors differing in ATP binding affinities may be involved. To specifically address the involvement of purinergic receptors on the ATP-mediated salvage effect, we added suramin as a universal P2-receptor inhibitor (Figure 4B) or the selective P2×7 inhibitor A-438079 (Figure 4C). Both inhibitors increased the ATP-mediated counter regulatory effect on Vac-induced cell death. These findings imply that purinergic signaling does not contribute to the observed ATP-related counter regulatory effect. Similar to arguments communicated in a recent study by Noerenberg et al. [13], we addressed the ubiquitously expressed TRPM7 to explain the ATP-mediated salvage effect on Vac-induced cell death. To prove the involvement of TRPM7, we applied carvacrol as a reversible TRPM7 inhibitor.

### Carvacrol supports Vac-induced cell death and prevents the ATP-mediated recovery effect on Vac

**Figure 5:**
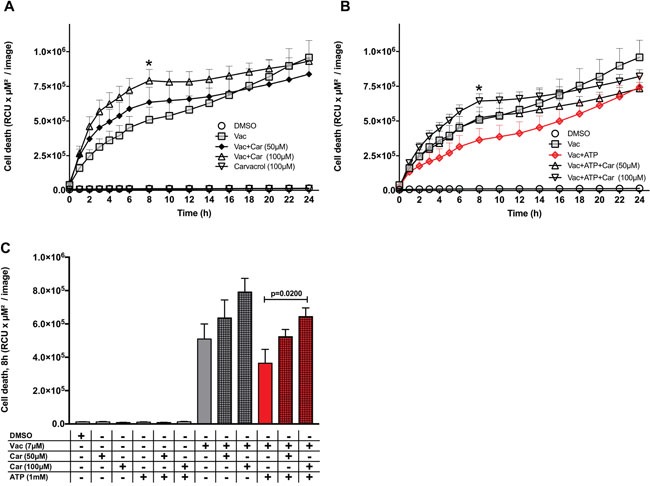
Carvacrol influences the ATP-recovery effect on Vac-induced cell death Semi-confluent #12537-GB cells (seeded in 96-well flat-bottomed microtiter plates) were treated with DMSO diluted in medium at 10^−4^ served as control, Vac (7 μM), Vac+ATP or ATP without carvacrol or with 50 μM and 100 μM carvacrol. PI-positive (dead) cells are given as RCU (y-axis, RCU×μM^2^/image). Glioma cells were followed for 24 h (x-axis) (IncuCyteZOOM®) **(A, B)**. Quantitative analysis at 8 h for glioma cell lines (* in A and B) **(C)**. Vac+ATP vs. Vac+ATP+carvacrol 100 μM (multiple comparison two way ANOVA, p=0.02). All values are given as means ± SD (6× replicates). All imaging was performed using IncuCyteZOOM® at 10× objective. Results are representative of 3 independent experiments.

In our approach to test the involvement of TRPM7, we quantified its expression by quantitative real-time PCR (qPCR) in glioma cell lines (#12537-GB, U-87) as well as in DPSCs. Accordingly, all cell lines expressed TRPM7, but expression in glioma cell lines (#12537-GB, U-87) was stronger than in dental stem cells (DPSCs) (Supplementary Figure 5). Moreover, the Vac-induced cell death and the observed ATP-mediated inhibitory effect on Vac-induced cell death were sensitive to carvacrol when co-incubated at 50 μM and 100 μM (Figure 5A). Cell death was monitored from 0–24 h. Carvacrol at 100 μM alone did not affect baseline glioma cell viability, but significantly stimulated 7 μM Vac-induced glioma cell death (two-way ANOVA, p<.0.0001: Vac/Vac+carvacrol 100 μM; Figure 5A). Moreover, 50 μM and 100 μM carvacrol impaired the ATP- (1 mM) mediated recovery effect on Vac-induced cell death (two-way ANOVA, p<0.0001) (Figure 5B). Figure 5C summarizes the quantitative differences on glioma cell death of Vac and the influence of ATP (1 mM) and carvacrol (50 μM and 100 μM) after 8 h, the time of greatest increment values. Because carvacrol blocks TRPM7, these results suggest that the ATP effects were mediated through TRPM7.

### Hypothetical mechanism of action

**Figure 6:**
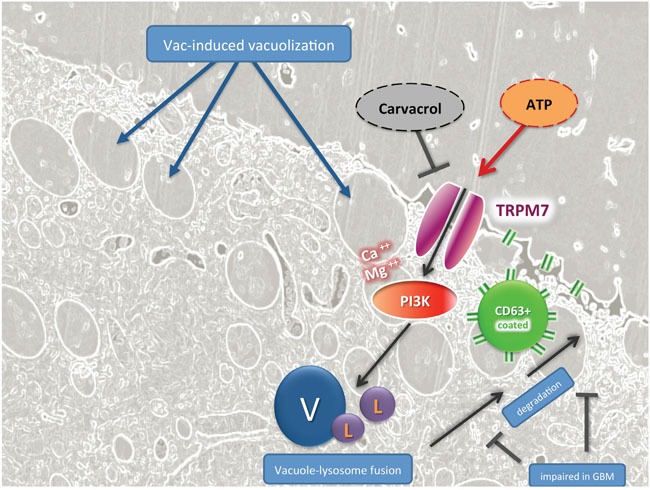
TRPM7-guided signaling in ATP-mediated counter regulation of Vac-induced cell death in GBM, hypothetical mode of action Fluorescence-based light microscopy and trans electron-microscopical imaging showed that Vac leads to massive vacuolization (V) related to macropinocytosis and autophagy, followed by cell death. This cartoon hypothesizes that exogenous ATP may activate Ca-signaling by TRPM7 activation, Ca^++^and Mg^++^ influx and PI3K activation, which may promote vesicle fusion (V) with lysosomes (L), and subsequent degradation by CD63-targeted vesicle extrusion. TRPM7-specific effects by exogenous ATP are sensitive to carvacrol, an effective inhibitor of TRPM7-related signaling in GBM.

According to our current working hypothesis, which is schematically displayed in Figure 6, Vac induces massive vacuolization (V) followed by cell death, which is due to either inefficient vacuole-lysosome fusion or impaired degradation and extrusion of vacuole contents by the CD63 vesicles. Ion-flux induced by exogenous ATP in turn induces calcium-signaling in Fura-2 labeled #12537-GB cells (Supplementary Figure 6). Exogenous ATP activates TRPM7, Ca^++^/Mg^++^ influx, and phosphoinositide 3-kinase (PI3K) activation, which promotes vesicle fusion (V) with lysosomes (L), and subsequent degradation by CD63-targeted extrusion. TRPM7-specific effects are sensitive to carvacrol, which stimulates TRPM7-related cell death in GBM.

## DISCUSSION

The present investigation documents rapid Vac-induced cell death in the commercially available U-87 cell line and the highly malignant glioma cell line #12537-GB, whereas non-malignant DPSCs remained unaffected. These findings mirror observations by Kitambi and colleagues on the highly specific action by Vac on GBM using established cell lines and their own isolates, but without affecting mouse embryonic stem cells, fibroblasts, mouse astrocytes and neurons [8]. The live cell imaging system IncuCyteZOOM® serves as a powerful tool for observing cell death in glioma cell lines, both morphologically and quantitatively (Figures 1, 2, 3, 4, 5). Vac-induced cell death is clearly independent from apoptosis [8] and displays intensive cell blebbing followed by immediate plasma-membrane rupture and subsequent PI uptake (Figures 1, 3 and Supplementary Figure 2), the latter is more a feature of necrosis [22]. This novel form of cell death was recently termed as methuosis [23]. These observations correspond with previous findings described by Kitambi et al. [8].

Cell death determined by PI-stained nuclei is significantly delayed in the presence of exogenous ATP. When we determined cell proliferation by confluence measurements (Supplementary Figure 7), the effect of ATP appeared to be independent from increased proliferation. However, in a different study, a growth-promoting effect by ATP was reported in gliomas [14], which makes additional investigations necessary to address ATP-responsive receptors and pathways in GBM. Because Vac likely acts similarly to small molecules of the chalcone family, which also mediate methuosis in GBM, a similar pathway may be involved [23–26]. According to Overmeyer and Maltese, methuosis induced by chalcones is Ras dependent [26] but the action profile of Vac has not been tested for Ras dependency. However, Kitambi and colleagues demonstrated that methuosis induced by Vac is MKK4 dependent [8], downstream of Ras/Rac-1 activation. In summary, further experiments need to address Ras/Rac-1/MKK4 signaling induced by both Vac and chalcones and its role in transformed and non-transformed cells. We here demonstrated that Vac is non-toxic against non-transformed DPSCs (Figure 1C), a potent regenerative stem-cell source [27]. Results confirmed previous observations regarding non-toxic effects on non-transformed tissues by Vac [8, 23]. Ras/Rac-1 and eventually MKK4 signaling may constitute a GBM-specific weak point exploited by these drugs. Overexpression of Rac-1 in GBM is likely to affect phagosome-lysosome fusion/degradation as schematically shown in Figure 6 [21, 22]. Accordingly, the GBM-specific deficiency in late phagosome (CD63^+^) [29] degradation results in methuosis by increased vacuole fusion events. Hypothetically (Figure 6), the ATP-recovery effect is mediated by purinergic receptors or TRPM channel family members, leading to Ca^++^ and Mg^++^ influx and PI3K activation. The latter may improve phagosome maturation and degradation [30, 31], and thereby attenuate the cytotoxic effect of Vac.

Vac-induced methuosis in GBM (#12537-GB and U-87) also involves caspase 3/7, as demonstrated in Figure 2, corresponding to results of Overmeyer and colleagues [23, 24]. In detail, caspase 3 activation was restricted to glioma cells already being detached from the surface [24]. However, caspase 3/7 is not essential for methuotic cell death [8, 26]. According to Kitambi and colleagues, Vac does not induce caspase 3/7 activation [8]. By contrast, we found that Vac induces caspase 3/7 activation (Figures 2, 3). In our caspase 3/7 assays (Promega chemiluminescent assay and IncuCyteZOOM®-based video microscopy), we analyzed caspase 3/7 activity in both attached and detached cells. One possible explanation for our different findings regarding Vac-induced caspase 3/7 activity could be that Kitambi and colleagues excluded the detached cells in their caspase assays. Although caspase 3/7 is not essential for methuotic cell death [8, 22], we found that caspase 3/7 activation as well as endogenous caspase 3/7 activity is counter regulated by exogenous ATP (Figure 2). In the glioma cell line #12537-GB, Vac-induced caspase 3/7 activation and STS-induced apoptosis occur within a similar time frame (Figures 2, 3, [32, 33]). However, the number of PI-positive nuclei in STS- and Vac-treated glioma cells differed between Vac- and STS-induced cell death (Figure 3A). Nuclear fragmentation, a characteristic of apoptotic cell death, was not observed in Vac-treated #12537-GB cells (Figures 3, Supplementary Figure 2), corresponding to observations by Overmeyer and colleagues [26]. Taken together, Vac induces a necrotic-like cell death (methuosis) through sharing vacuolization and caspase 3/7 activation (but no other features) characterizing apoptotic cell death. Caspase-3 activation has been reported to be involved in autophagic cell death [34]. Of note, autophagic cell death features vacuolization [35] and plasma-membrane rupture [36], considerable resembling the observation reported herein for Vac-induced cell death in GBM. However, whether autophagy plays a role in Vac-induced cell death awaits further experiments.

Titration of different ATP concentrations into #12537-GB Vac assays antagonized Vac-induced cell death already from only 1 μM ATP (Figure 4A). By contrast, at lower concentrations, addition of ATP had little if any effect (100 nM ATP), or even increased (10 nM ATP) Vac-induced cell death (Figure 4A).

High ATP concentrations (from 1 μM up to 1 mM) have been reported to occur *in vivo* in the extracellular space under pathophysiological conditions, including hypoxia [37]. Our findings suggest the contribution of different ATP receptors with distinct ATP affinities in preventing or increasing the Vac-induced cell death. P2×4 and P2×7 have been identified in #12537-GB (Supplementary Figure 4).

ATP at 1–10 μM potentially activates P2×1, P2×2, P2×3, P2×4, P2×5 and P2×6, whereas P2×7 possesses lower affinity (EC_50_>100 μM) [12, 38]. In addition to the ionotropic P2X receptors, ATP acts as an agonist on the metabotropic P2Y receptors: P2Y2, (EC_50_=100 nM), P2Y11 (EC_50_=17 μM) and P2Y13 (EC_50_=260 μM) [38]. To address the involvement of purinergic receptors, we applied 30 μM suramin, a nonselective potent inhibitor of P2 receptors [12, 14] and the selective P2×7 inhibitor A-438079 [12]. Both inhibitors failed to impair the recovery effect of 1 mM ATP on Vac-induced cell death (Figure 4B, 4C, respectively). By contrast, these inhibitors even increased the ATP-mediated recovery effect on Vac-induced cell death. These findings suggest that purinergic signaling does not contribute to the observed ATP-related counter regulatory effect at 1 mM ATP. Indeed, the simple observation that a complete salvage effect by ATP (TRPM7 mediated) could not been obtained can be explained by the function of purinergic receptors. This explanation is supported by experiments performed in the presence of suramin or A-438079 (see above, Figure 4B, 4C).

Overall, the ATP-inducible and carvacrol-sensitive ion channel TRPM7 plays a major role in Vac-induced methuosis (Figure 5), as exemplified by Chen et al. [19]. TRPM7 is frequently overexpressed in malignant cells as well as in our glioma cell lines (Supplementary Figure 5). Activation by exogenous ATP [13] stimulates the influx of divalent metal ions (e.g. Ca^++^ and Mg^++^) [39, 40], which is essential for mammalian Mg^++^ homeostasis [41]. Recently, an important influence of TRPM7-mediated Mg^++^ influx on PI3K activity was reported by Sahni and Scharenberg [42]. Because PI3K activation leads to improved endosomal trafficking (Figure 6, [30, 31]), this may at least in part explain the ATP-mediated recovery effect on Vac-induced methuosis. Indeed, inhibition of the observed ATP-mediated inhibitory effect on the Vac-induced cell death by carvacrol emphasizes the involvement of TRPM7 (Figures 5, 6) [19]. Further compounds that inhibit TRPM7 are currently being investigated to further confirm the role of TRPM7 in methuosis [43].

Vac induces a dramatic cell death featuring rupture of the plasma membrane, termed methuosis. Extracellular ATP, an important danger signal in cancer [20], might limit Vac-induced cell death when Vac is applied *in vivo*. A remarkable feature of GBM with a highly unfavorable outcome [9] are the large necrotic foci [44], which may be a source of extracellular ATP [11, 45]. Therefore necrotic areas constitute a potentially important prognostic factor to be considered for the development of Vac-based treatment protocols in future.

In conclusion, Vac induces a non-apoptotic cell death despite caspase 3/7 activation in glioma cells but does not affect the viability of multipotent DPSCs. According to our findings, Vac-induced cell death is highly efficient when compared to established apoptosis inducing drugs like Temozolomide. However, methuosis induced by Vac is sensitive to extracellular ATP likely mediated by TRPM7 activation. Accordingly, TRPM7 inhibition may be considered as an important therapeutic option improving Vac-induced methuosis once Vac enters the clinical phase. Although promising, such *in-vitro* studies remain limited unless an appropriate transfer to a clinically relevant model can been achieved.

## MATERIALS AND METHODS

### Cell lines and cell culture

The glioma cell line #12537-GB was established from primary tumor material as described below (approved by the local Ethics Committee of the University Hospital Ulm; universal trial number: U111-1179-3127) with patient-informed consent. The tumor material was minced and cells from the tumor material were taken into culture by trypsinization of the tumor material (2.5% trypsin), followed by Ficoll separation. Continuous cultures were performed in Iscove's Modified Dulbecco's Medium (IMDM) (Lonza.com, USA) supplemented with 10% fetal calf serum (FCS, endotoxin-free, Batch 0247x, Merck/ Biochrom.com, Germany), GlutaMAX (ThermoFisher.com, USA) and antibiotics at 37°C under 5% CO_2_. Two volunteers donated their third molars to establish DPSCs. Pulpa tissue was mechanically dissected and trypsinized from the third molars, followed by culture in IMDM containing 10% endotoxin-free FCS (Batch No.: 0247x, Merck/ Biochrom.com), further details were according to Cvicl et al. [46].

The glioma cell line #12537-GB (passage >100) was established from a 63 year-old male patient diagnosed with temporo-parietal (right) localized primary GBM (World Health Organization Grade IV) with a sarcomatous component, isocitrate dehydrogenase 1/2 wild type, O6-methylguanine-DNA methyltransferase not methylated and with multiple chromosomal aberrations. The tumor material was derived from a relapse (localization tempero-occipital, left), which occurred 5 months after surgical resection followed by adjuvant radiochemotherapy (3 cycles Temozolomide, radiation: 60 Gy). The recurrent tumor (WHO grade IV) displayed necrotic regions characteristic for GBM. The phenotype of the cell line derived from this tumor material was assessed by flow cytometry comprising surface (Supplementary Table 1) and cytoplasmic staining (Supplementary Table 2). Results were compared with the commercially available glioma cell line U-87 (ATCC.org) and DPSCs (passage 3) established in this laboratory.

### Experiments using the IncuCyteZOOM® incubator microscope

For stimulation experiments, cells were trypsinized and seeded into transparent flat-bottom 96-well plates (NUNC) at a seeding density of 5,000–cells/well. For IncuCyte experiments, cells were cultured in phenol red and riboflavin-free FluoroBrite Dulbecco's Modified Eagle Medium (DMEM) medium (ThermoFisher.com) supplemented with 10% FCS, GlutaMAX and antibiotics at 37°C under 5% CO_2_. Vac (Sellekchem.com, Germany) was dissolved in dimethyl sulfoxide (DMSO) (Sigma.com, USA) to a concentration of 76 mM (155 μl were added to 5 mg Vac). ATP (Invivogen.com) was dissolved in sterile water to a concentration of 100 mM. Dilutions were made using FluoroBrite DMEM medium. Semi-confluent cell layers seeded into 96-well flat-bottomed microtiter plates (ThermoFisher.com) were treated with a final concentration of 7 μM Vac in the presence of 10 μg/ml PI (Sigma.com) with and without ATP, carvacrol (Sigma.com), suramin (Tocris/Biotechné.com, UK) or A-438079 (Sellekchem.com). Controls were performed with 10^−4^ DMSO diluted in medium no toxic effects on glioma cell lines or DPSCs.

After adding the reagents, the plate was inserted into the IncuCyteZOOM® (Essenbio.com, USA). Cell death was quantified as the mean total red integrated fluorescence intensity per image (relative color units (RCU) × μM^2^/image). Data were collected and visualized using GraphPad Prism 7 software. The kinetics of caspase 3/7 activities were determined by the IncuCyte caspase 3/7 assay (http://www.essenbioscience.com/en/products/reagents-consumables/incucyte-96-well-kinetic-caspase- 37-apoptosis-assay-kit/ Caspase 3/7 activity is presented graphically as the mean total green integrated fluorescence intensity (RCU × μM^2^/image).

### Caspase-Glo® 3/7 assay

Cells were seeded into white non-transparent LumiNunc 96-well flat-bottomed microtiter plates (ThermoFisher.com) at a density of 5,000 cells per well and treated with the respective Vac and ATP concentrations following incubation for 2–6h at 37°C under 5% CO_2_. Thereafter, plates were adjusted to room temperature and Caspase-Glo® 3/7 reagent was added to all wells. Luminescence was determined after 30 min or longer according to the manufacturer's protocol (Promega.com, USA). The read-out of the luminescence signal (RLUs) was obtained using a GloMax® 96 Microplate luminometer (Promega.com).

### Phenotype of #12537-GB: P2×1, P2×4 and P2×7

P2×1 (APR-001), P2×4 (APR-002) and P2×7 (APR-004) antibodies were purchased from Alomone (Alomone.com, Israel). For flow cytometry, cells were detached using accutase (Sigma.com). For each staining tube, 1×10^4^ cells were used. Cells were fixed and permeabilized using Perm/Fix (BDbiosciences.com, USA), followed by two washes with Perm/Wash (BDbiosciences.com) and stained using the respective antibodies, washed with sodium azide buffer (0.01% NaN_3_ and 0.025% FCS) and fixed with BD CellFix (BDbiosciences.com). For P2×1 and P2×4, cells were stained using secondary antibody (goat anti-rabbit antibody (IgG F(ab’)_2_ fragment adsorbed for human proteins), Rockland.com, USA). Secondary antibody staining alone served as negative controls. Ten thousands cells were counted with a FACSCalibur (BDbiosciences.com). Evaluation was performed using Cellquest software v5 (BDbiosciences.com) and FlowJo software v10 (FlowJo.com, USA).

### Flow cytometry for glioma cell lines and DPSC

For cytoplasmic staining, cells were fixed and permeabilized using Perm/Fix (BDbiosciences.com), followed by two washes with Perm/Wash (BDbiosciences.com) and stained for 20 min using the respective antibodies (Supplementary Table 2, 3), washed with sodium azide buffer and fixed with BD CellFix (1×). For surface staining, cells were detached using accutase (Sigma.com) and stained with the respective antibodies (Supplementary Table 1, 3), washed with sodium azide buffer and fixed with BD CellFix (1×). Ten thousands cells were counted with a FACSCalibur (BDbiosciences.com). Evaluation was performed using Cellquest software (BDbiosciences.com). Mean fluorescence intensity (MFI) values were calculated using the following formula: % positive of gated cells x X-mean or Y-mean.

### Quantitative real-time PCR (qPCR)

Total RNA was extracted using the Maxwell® 16 LEV simply RNA kit (Promega.com) according to the manufacturer's instruction. In total, 400 ng RNA were used for cDNA first-strand synthesis using the GoScript Reverse Transcription System (Promega.com) according to the manufacturer's instruction. cDNA was diluted 1/10 for qPCR; 4 μl were used for qPCR. qPCR was performed using TaqMan Gene expression assays (RPL13: Hs00744303_s1, TRPM7: Hs00559080_m1) and a StepOne System (ThermoFisher.com) according to the manufacturer's protocol. PCR conditions: 2min 50°C, 10min 95°C followed by 40 cycles comprising 15s 95°C (denaturation) and 1min 60°C (annealing/extension). Data are presented as fold change (Relative Quantity (RQ)) relative to RPL13 as endogenous control. DPSCs were used as the reference sample. Technical triplicates were performed.

### Electron microscopy

Sapphire discs (Engineering Office M. Wohlwend GmbH, Switzerland) were used as support for the adherent glioma cell culture. The 0.170-mm thick discs were cleaned by sonication for 15 min each in 60% sulfuric acid, soapy water and twice in absolute ethanol, and allowed to dry. The discs were then coated with approximately 20 nm carbon by electron-beam evaporation and dried for 8 h or overnight in an oven at 120°C to increase the carbon-film stability. The coated sapphire discs were then carefully immersed in complete medium and the cells were seeded on top. A 50-μm gold spacer ring (diameter 3.05 mm, central bore 2 mm; Plano GmbH, Germany) was mounted in between two coated sapphire discs with the cells grown on them, similar to the protocol introduced by Hawes et al. [47]. These sandwiches were high-pressure frozen without aluminum planchettes and without the use of hexadecene. Freeze substitution was performed as described in Walther and Ziegler [48] and Buser and Walther [49] with a substitution medium consisting of acetone with 0.2% osmium tetroxide, 0.1% uranyl acetate and 5% water for 19 h. During this time period, the temperature was exponentially raised from 183 K to 273 K. After substitution, the samples were maintained at room temperature for 30 min and then washed twice with acetone. After stepwise embedding of the samples in Epon (Fluka.com, USA) (polymerization at 333 K within 72 h), they were cut using a microtome (Leica Ultracut UCT ultramicrotome) with a diamond knife (Diatome, Switzerland) to semi-thin sections with a nominal thickness of 500 nm or 1 μm, as measured from the readout of the microtome. Specimens were examined using a JEOL 1400 (JEOL.com, Germany) at 120 keV.

### Statistical analysis

Statistical analysis was performed using GraphPadPrism v7 (Graphpad.com, USA). To identify significant differences between treatments over the entire time frame, regular two-way ANOVA was performed. P-values of single time points of kinetics were calculated by multiple comparison tests after two-way ANOVA. Sidak's correction was applied to correct for multiple comparisons. P-values of caspase 3/7 Glo assays were calculated using unpaired two-tailed t-test with Welch's correction. Observations with p<0.05 were considered as significant.

## SUPPLEMENTARY MATERIALS FIGURES AND TABLES



## Abbreviations

Car: Carvacrol
DPSCs: Dental-pulp stem cells
GBM: Glioblastoma multiforme
GFAP: Glial fibrillary acidic protein
MFI: Mean fluorescence intensity
MIC-A: MHC class I polypeptide-related sequence A
RCU: Relative color units
RLU: Relative luminescence units
STS: Staurosporine
Vac: Vacquinol-1
VEGF: Vascular endothelial growth factor
zVAD-FMK: Carbobenzoxy-valyl-alanyl-aspartyl-[O-methyl]-fluoromethylketone
