# Supplementary material for: Vacquinol-1 inducible cell death in glioblastoma multiforme is counter regulated by TRPM7 activity induced by exogenous ATP
Source: Oncotarget. 2017 Mar 30;8(21):35124–37. doi: 10.18632/oncotarget.16703 (PMC5471040; doi:10.18632/oncotarget.16703)
Supplement: Supplementary file 1 [file oncotarget-08-35124-s001.pdf]

Vacquinol-1 inducible cell death in glioblastoma multiforme is counter regulated by TRPM7 activity induced by exogenous ATP

SUPPLEMENTARY MATERIALS

SUPPLEMENTARY FIGURES AND TABLES

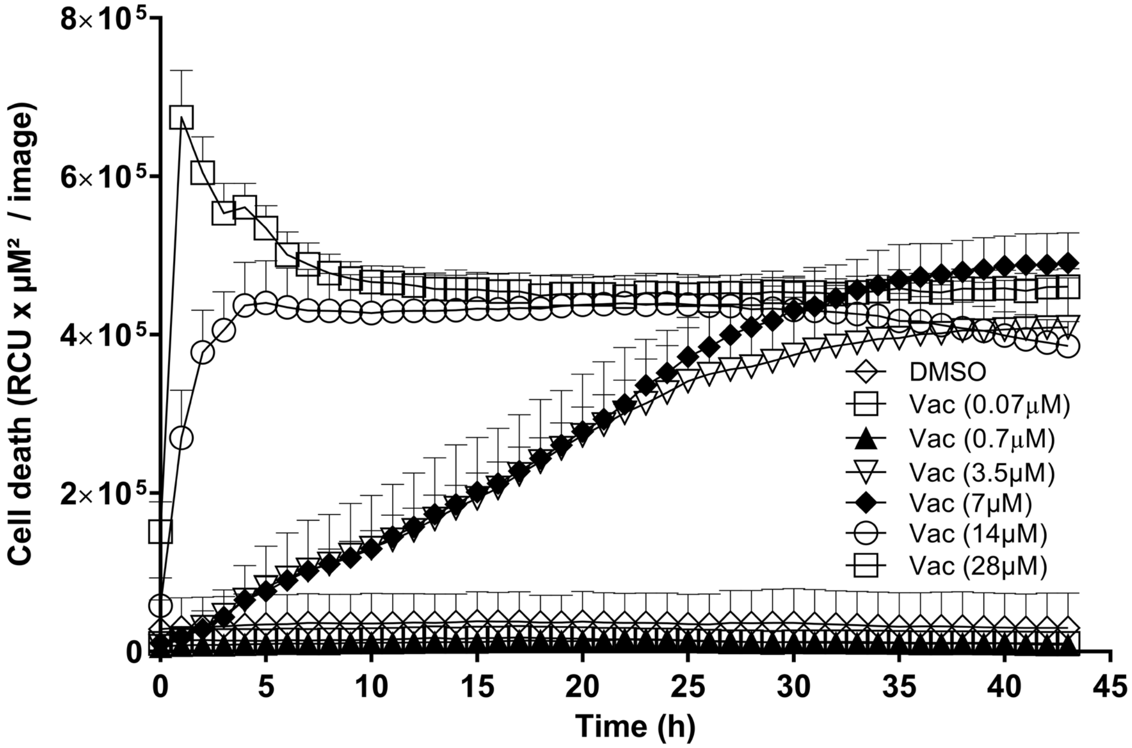

Supplementary Figure 1: Titration of Vac. #12537-GB cells were treated with DMSO (diluted to 2.5× 10^{-3} in medium, control); Vac: 0.07 µM; 0.7 µM; 3.5 µM; 7 µM; 14 µM; 28 µM. PI-positive (dead) cells are given as RCU (y-axis, total red integrated fluorescence intensity) per µM²/image. Glioma cells were followed for 43 h (x-axis). All values are given as means ± SD (n=3). All imaging was performed using IncuCyteZOOM at 10× objective.

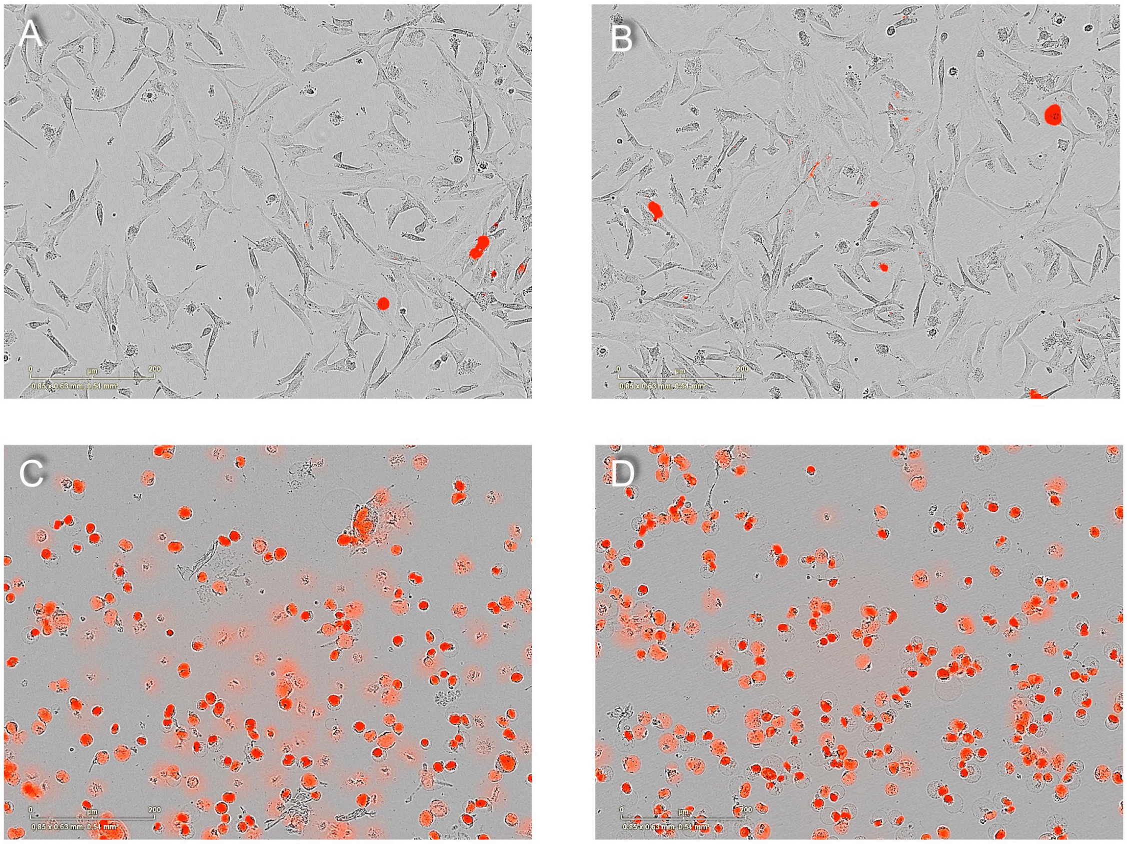

Supplementary Figure 2: Vac-induced cell death differs morphologically from apoptosis. Semi-confluent glioma cells were treated with DMSO (diluted to 10^{-4} in medium, control). Representative images of two independent experiments **(A, B)**, and Vac (7 µM) of two independent experiments **(C, D)**. Dead cells were stained using PI after 24 h; images were obtained using IncuCyteZOOM at 20× objective. All images are phase-contrast merged with red fluorescence. Necrotic nuclei stain pale-red with PI and condensed nuclei stain bright-red with PI. Relative quantities may differ.

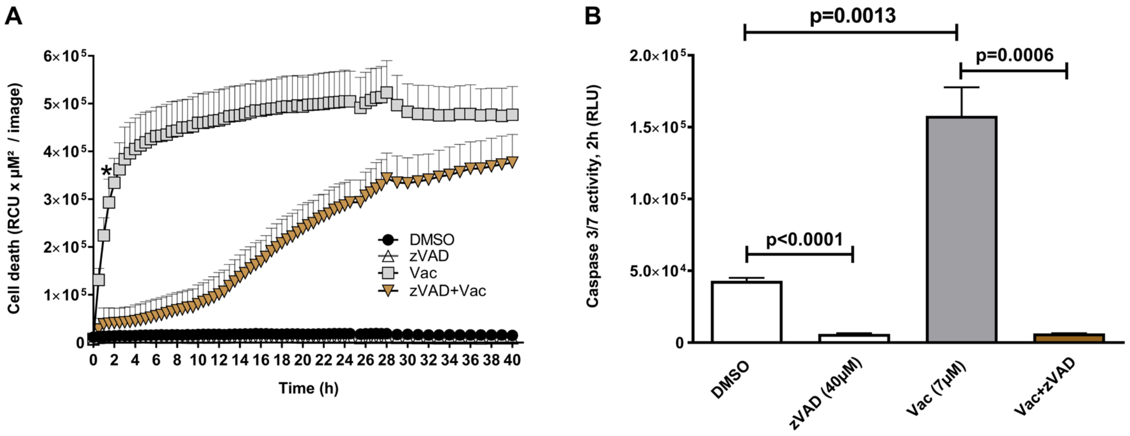

Supplementary Figure 3: Vac-induced cell death is only partially sensitive to caspase-inhibition. Semi-confluent #12537-GB cells (seeded in 96-well flat-bottomed microtiter plates) were treated with 7 µM Vac with or without 40 µM zVAD-FMK, or DMSO diluted in medium at 0.2% served as control. PI-positive (dead) cells are given as RCU (y-axis, total red integrated fluorescence intensity) per µM²/image. Glioma cells were followed for 40 h (x-axis). All values are means of PI fluorescence ± SD (6× replicates) **(A)**. All imaging was performed using IncuCyteZOOM® at 20× objective. Caspase 3/7 activity was determined by luminescence assay (RLU, y-axis by Caspase Glo 3/7 assay, Promega.com) after 2 h (*, A); control vs. zVAD-FMK (t-test, $p<0.0001$); control vs. Vac (t-test, $p=0.0013$); Vac vs. Vac+zVAD (t-test, $p=0.0006$). All values are means of RLU with background subtracted ± SD (4× replicates) **(B)**.

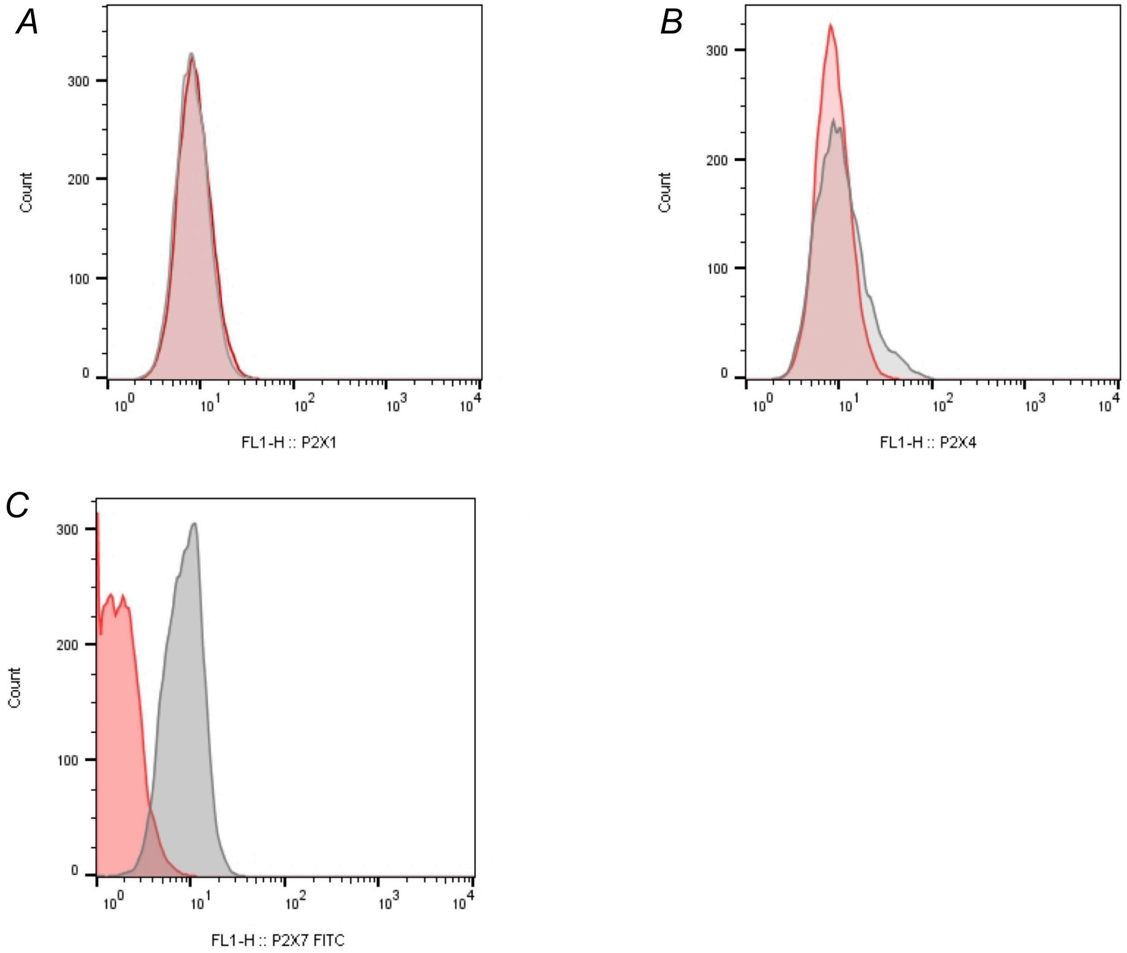

Supplementary Figure 4: Expression analysis of P2X1, P2X4 and P2X7 in #12537-GB. Antibodies directed against P2X1 and P2X4 (rabbit polyclonal, Alomone), detected by secondary antibodies (goat-anti rabbit, F(ab)’$_2$), and P2X7 FITC (rabbit polyclonal) are shown as histograms (grey) compared to the negative control (red). P2X1 **(A)**, P2X4 **(B)** and P2X7 **(C)**. Histograms were created by FlowJO software (Version 10).

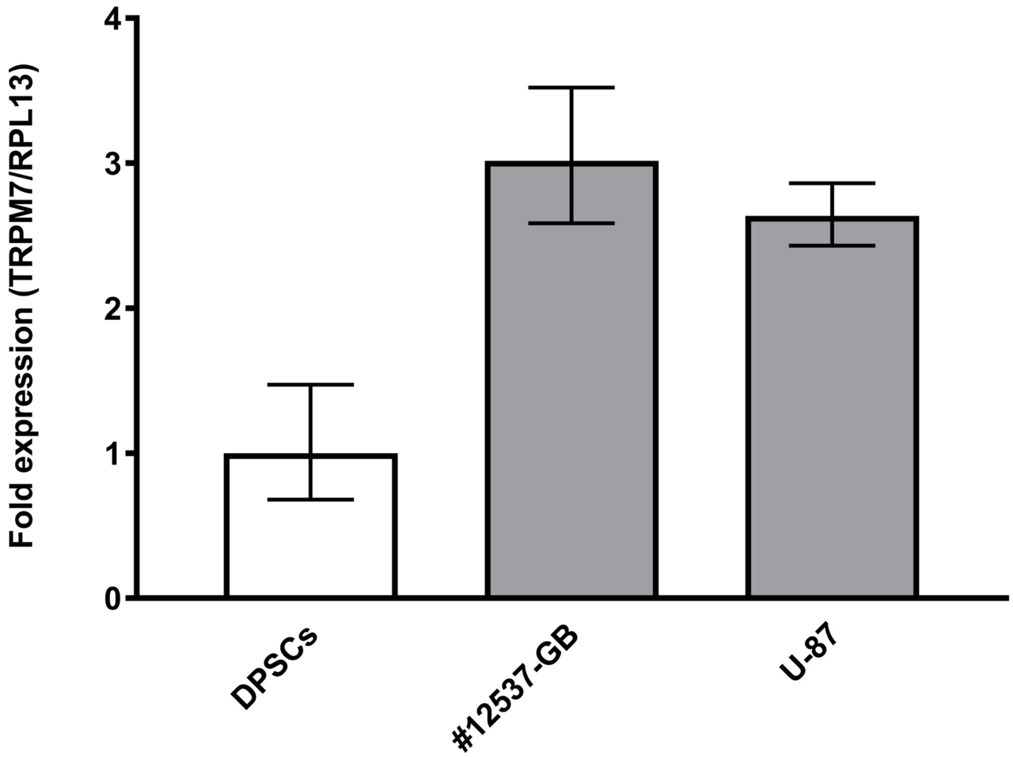

Supplementary Figure 5: TRPM7 is expressed in DPSCs and glioma cell lines, mRNA from DPSCs, #12537-GB and U-87. RNA was prepared by Maxwell® LEV 16 simplyRNA kit (Promega.com) and first-strand cDNA synthesis was performed using GoScript Reverse transcription system (Promega.com). TRPM7 mRNA in DPSCs, #12537-GB and U-87 cells was detected by qPCR and normalized to RPL13. All values are means of fold expression (RQ, Relative Quantity values) normalized to DPSCs as control (technical triplicates). Error bars indicate the range between the lowest and the highest RQ values.

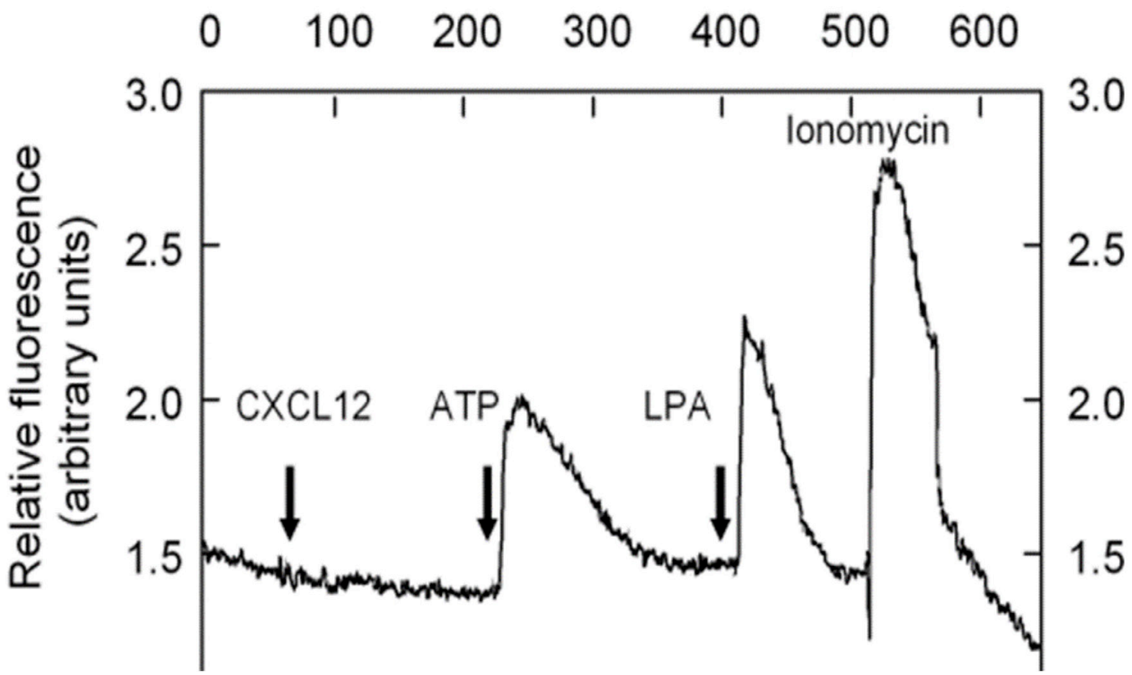

Supplementary Figure 6: Calcium signaling in #12537-GB. Calcium responsiveness was tested in Fura-2 (Thermofisher.com)-labelled glioma cells using a LS55 Luminescence Spectrometer (PerkinElmer.com). After labeling, cells were diluted in HBSS (Hanks balanced salt solution) at a cell concentration of $1x10^6$ per ml. Cells were stimulated with rhCXCL12 (100 nM, PeproTech.com), ATP (1 mM), Lysophosphatidic acid (LPA, 20 µM, Sigma.com) and ionomycin (1 µM, Sigma.com). Results are given as relative fluorescence (arbitrary units).

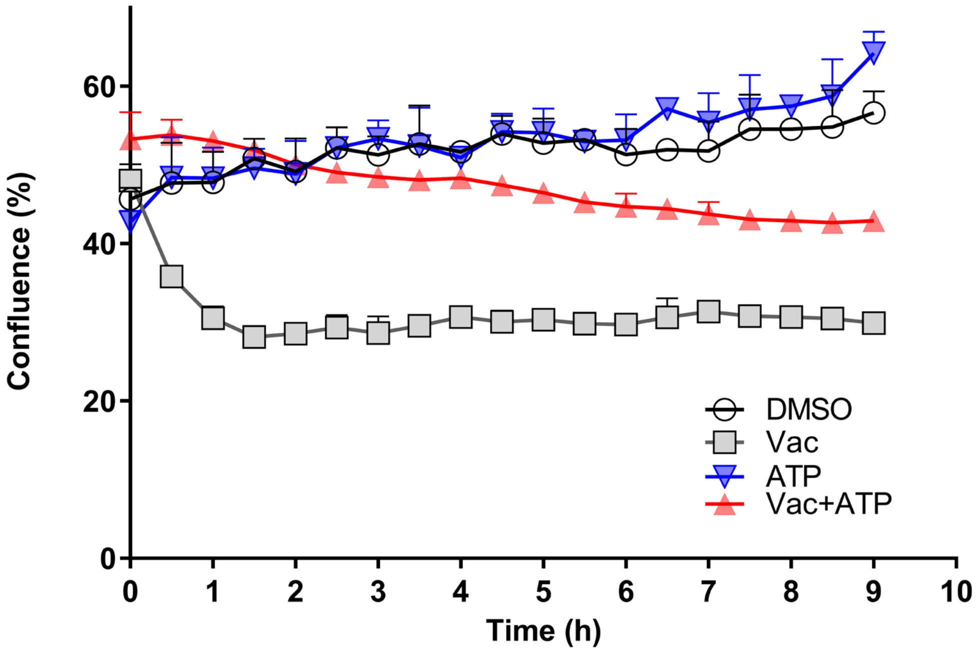

Supplementary Figure 7: Vac leads to a decrease in confluence, which is counter regulated by exogenous ATP. #12537-GB cells were treated with DMSO (diluted to 10^{-4} in medium, control); Vac 7 µM; Vac+ATP 1 mM; and 1 mM ATP alone and monitored for 9 h (x-axis). Confluence is given as % (y-axis). All values are given as means ± SD (n=2). All imaging was performed using IncuCyteZOOM at 10× objective. Results are representative for 2 independent experiments.

Supplementary Table 1: Surface antigens in glioma cell lines and DPSCs

Marker	#12537-GB [%] *	#12537-GB [MFI]	U-87 [%]	U-87 [MFI]	DPSCs [%]	DPSCs [MFI]
HLA class I	99.7	100,834.1	96.4	6,337.6	99.66	52,858.4
HLA-DR	6.3	328.4	30.6	492.8	0.0	0.0
CD39	0.0	0.0	0.0	0.0	0.0	0.0
TRAIL-DR5	74.5	3,026.4	69.5	1,500.9	0.0	0.0
CD74	0.7	134.2	2.7	58.7	9.79	347.1
CD63	96.2	16,468.8	97.3	11,051.3	57.3	1,627.8
CD178	0.0	0.0	0.0	0.0	0.0	0.0
CD146	2.6	72.8	33.0	1,134.8	99.69	42,202.8
CD163	0.0	0.0	0.0	0.0	0.0	0.0
CD31	0.0	0.0	0.0	0.0	1.79	97.4
CD155	56.5	1,793.9	92.9	2,612.6	0.0	0.0
CD56	0.0	0.0	0.0	0.0	0.0	0.0
MIC-A	5.4	119.1	37.1	716.4	6.31	211.8
CD54	4.7	124.9	10.4	146.3	0.0	0.0
CD95	89.5	4,731.2	49.7	995.4	93.04	6,144.4
GranzymeB	0.0	0.0	0.0	0.0	0.0	0.0

Surface expression of MHC, TRAIL-DR5, MIC-A, granzyme B and CD antigens in #12537-GB, U-87 and DPSCs (passage 3) determined by flow cytometric analysis. Data expressed as percent positive cells and MFI.

Supplementary Table 2: Cytoplasmic antigen expression by glioma cell lines and DPSCs

Marker	#12537-GB [%] *	#12537-GB [MFI]	U-87 [%]	U-87 [MFI]	DPSCs [%]	DPSCs [MFI]
CD133	0.0	0.0	0.0	0.0	0.0	0.0
GFAP	74.4	2,336.2	63.0	4,791.0	85.69	116.5
S100-A8	40.5	1,008.3	0.0	0.0	0.0	0.0
VEGF	0.0	0.0	0.0	0.0	0.0	0.0
Arginase-1	54.9	2,737.9	77.1	3,020.9	82.0	6,211.5

Cytoplasmic expression of CD133, GFAP, S100-A8, VEGF, and Arginase-1 (Arg-1) in #12537-GB, U-87 and DPSCs (passage 3) determined by flow cytometric analysis. Data expressed as percent positive cells and MFI.

Supplementary Table 3: Antibodies applied for phenotypical analysis in glioma cell lines and DPSCs

Marker	Clone	Provider
HLA class I	W6/32HL	Abcam (UK)
HLA-DR	L243	BD Biosciences (USA)
CD39	A1	BioRad (USA)
TRAIL-DR5	DJR2-4	eBioscience (USA)
CD74	5-329	Miltenyi (Germany)
CD63	CLB-180	ThermoFisher (USA)
CD178	Alf-2.1a	Ancell (USA)
CD146	P1H12	BD Pharmingen (USA)
CD163	5C6-FAT	Acris GmbH (Switzerland)
CD31	WM-59	eBioscience
CD155	PV404.19	Miltenyi
CD56	AF12-7H3	BD Biosciences
MIC-A	159227	R&D Systems (USA)
CD54	RR1/1	eBioscience
CD95	DX2	BD Biosciences
GrB	GB11	BD Pharmingen
CD133	293C3	Miltenyi
GFAP	1B4	BD Pharmingen
S100	CF-145	LsBio (USA)
VEGF	VG1	Acris GmbH
Arginase-1	Ref. P05089 (polyclonal)	R&D Systems
IgG 2b (isotype control)	27-35	BD Pharmingen
IgG1 (isotype control)	679.1Mc7	Immunotech (France)

Antibody designation, clone, and provider are displayed.
